# Association of Atrial Fibrillation with Incident Probable Dementia and Cognitive Impairment in the Systolic Blood Pressure Intervention Trial (SPRINT) †

**DOI:** 10.3390/jcm14134791

**Published:** 2025-07-07

**Authors:** Parham Samimisedeh, Richard Kazibwe, Christopher L. Schaich, Timothy M. Hughes, Elsayed Z. Soliman

**Affiliations:** 1 Epidemiological Cardiology Research Center, Section on Cardiovascular Medicine, Department of Medicine, School of Medicine, Wake Forest University, Winston-Salem, NC 27157, USA; parhamsamimimd@gmail.com; 2 Hospital Medicine, Department of Internal Medicine, School of Medicine, Wake Forest University, Winston-Salem, NC 27157, USA; rkazibwe@wakehealth.edu; 3 Hypertension and Vascular Research Center, School of Medicine, Wake Forest University, Winston-Salem, NC 27157, USA; 4 Gerontology and Geriatric Medicine, Department of Internal Medicine, School of Medicine, Wake Forest University, Winston-Salem, NC 27157, USA; tmhughes@wakehealth.edu

**Keywords:** atrial fibrillation, blood pressure, hypertension, dementia, cognitive impairment, SPRINT tria

## Abstract

**Background:** The association between atrial fibrillation (AF) and dementia and cognitive decline in individuals with hypertension is not well established. **Methods**: The Systolic Blood Pressure Intervention Trial (SPRINT) enrolled participants with hypertension at high risk of cardiovascular disease (CVD) but without diabetes or a history of stroke. Participants with baseline pre-existing clinical dementia, prescribed dementia medications, or missing AF or cognition data were excluded. AF was identified using centrally read electrocardiograms. Mild cognitive impairment (MCI) and probable dementia (PD) were determined during follow-up by an adjudication committee. Multivariable Cox proportional hazards regression models were employed to assess the association of time-dependent AF with MCI, PD, and a composite endpoint of MCI/PDI. **Results**: A total of 8539 participants (mean age: 67.9 years; 35.1% female) were included in the analysis. Of these, 264 had AF at baseline or during follow-up. Over a median follow-up period of 5 years, 318 PD, 625 MCI, and 849 composite PD or MCI events occurred. In models adjusted for treatment assignment, baseline sociodemographics, CVD risk factors, and potential confounders, time-dependent AF was associated with incident PD, MCI, and a composite endpoint of MCI/PDI [HR (95% CI): 1.84 (1.09, 3.13), 1.59 (1.01, 2.53), and 1.63 (1.12, 2.38), respectively]. Further adjustment for incident stroke did not significantly change these associations. **Conclusions**: AF is associated with an increased risk of dementia and cognitive impairment in patients with hypertension but not diabetes or stroke. Further research is needed to determine whether AF management strategies can mitigate cognitive decline.

## 1. Introduction

Thromboembolic adverse neurological outcomes are among the most common and clinically significant consequences of atrial fibrillation (AF). In patients with AF, poor cognitive outcomes have been attributed to transient ischemic attacks (TIA) and thromboembolic strokes [1,2]. Nevertheless, emerging evidence suggests that AF may lead to cognitive impairment and dementia in patients with or without a history of stroke, indicating potential mechanisms beyond thromboembolic origin [3,4]. However, the extent of this association remains unclear in patients with hypertension. Previous studies have indicated that each 1 mmHg increase in systolic blood pressure (SBP) is associated with a 1.8% increase in the relative risk of developing AF [5,6], and individuals with untreated hypertension have a 42% increased risk of dementia compared with healthy controls [7]. Whether AF is associated with cognitive decline and probable dementia (PD) in patients already at high risk is unclear. Therefore, we investigated the association between AF and cognitive impairment in hypertensive patients without stroke using data from the Systolic Blood Pressure Intervention Trial (SPRINT).

By focusing on a hypertensive, stroke-free population, this study addresses a critical knowledge gap by isolating the potential independent contribution of AF to cognitive decline, offering insights that may inform risk stratification and preventive strategies in a group already predisposed to vascular and neurodegenerative outcomes.

## 2. Methods

### 2.1. Data Availability

All data were sourced from the National Institutes of Health Biologic Specimen and Data Repository Information Coordination Center. Access to these resources is available at https://biolincc.nhlbi.nih.gov/studies/sprint/, accessed 20 December 2024.

### 2.2. Study Design and Participants

This was a post-hoc analysis of the SPRINT-Memory and Cognition in Decreased Hypertension (MIND) study, which was part of the SPRINT trial. The design and protocols of SPRINT and SPRINT-MIND have been previously described [8,9]. Briefly, SPRINT enrolled 9361 participants with hypertension but without diabetes mellitus, who were randomized to either a standard or intensive BP control group. The target SBP was set at <140 mmHg in the standard treatment group and <120 mmHg in the intensive treatment group. In SPRINT-MIND, the primary outcome was the incidence of adjudicated PD, with secondary outcomes including Mild Cognitive Impairment (MCI) and a composite measure of PD and MCI. The primary goal of the SPRINT-MIND study was to evaluate whether intensive SBP control reduces the incidence of all-cause dementia.

The exclusion criteria included known secondary hypertension; a history of TIA or stroke at baseline; symptomatic heart failure within the past six months or an ejection fraction below 35%; diagnosis of polycystic kidney disease (PKD), end-stage renal disease (ESRD), or estimated glomerular filtration rate (eGFR) < 20 mL/min/1.73 m^2^; and a baseline diagnosis of dementia or use of dementia-specific medications. A comprehensive list of exclusion criteria is provided in the SPRINT-MIND protocol [8]. For the current post-hoc analysis, participants with missing data on baseline and follow-up AF or cognitive outcomes were excluded from the study.

Institutional Review Board (IRB) approval was obtained from all study sites, and all participants provided written informed consent.

### 2.3. Electrocardiogram and Atrial Fibrillation Diagnosis

Electrocardiograms (ECGs) were recorded at baseline, two- and four-year follow-up visits, and close-out visits. Digital ECGs were captured using a GE MAC 1200 electrocardiograph set to a calibration of 10 mm/mV and a recording speed of 25 mm/s. All ECGs were centrally processed at the EPICARE ECG Center (Epidemiological Cardiology Research Center, Wake Forest University School of Medicine, Winston-Salem, NC, USA). AF was automatically detected using GE MUSE 12-SL Marquette (version 2001; GE, Milwaukee, WI, USA) and then visually confirmed by a cardiologist. Baseline ECG monitoring was used to determine AF status at baseline for all study participants. Incident AF cases were defined as new-onset AF diagnoses identified during subsequent ECG assessments conducted during the follow-up period.

### 2.4. Ascertainment of Probable Dementia and Mild Cognitive Impairments

Trained examiners evaluated cognitive status at baseline, at 2-year and 4-year follow-ups, and study close-out if it occurred more than one year after the 4-year follow-up. However, at the time of the early discontinuation of the SPRINT trial, many scheduled cognitive assessments for the 4-year period had not been completed. These assessments were subsequently conducted during extended follow-up visits.

The cognitive assessment protocol included tests of global cognitive function (Montreal Cognitive Assessment [MoCA]), learning and memory (Logical Memory Forms I and II from the Wechsler Memory Scale), processing speed (Digit Symbol Coding Test from the Wechsler Adult Intelligence Scale), and functional capabilities (Functional Activities Questionnaire). All available test results and questionnaire data were submitted to a centralized web-based platform for review and adjudication by an expert panel specializing in dementia. Based on standardized diagnostic criteria, two independent adjudicators who were masked to the treatment assignment classified the participants into three cognitive categories: no cognitive impairment, MCI, or PD. PD was defined based on the National Institute on Aging–Alzheimer’s Association (NIA-AA) core clinical criteria for probable Alzheimer’s disease dementia. This included (1) a gradual, insidious onset of symptoms progressing over months to years; (2) documented cognitive decline by informant report or clinical observation; and (3) prominent cognitive deficits in memory or other domains (e.g., language or executive function). Patients with substantial cerebrovascular disease, core features of other neurodegenerative syndromes, or medical or psychiatric conditions likely to affect cognition were excluded. MCI was also defined according to the NIA-AA criteria as a clinical and cognitive syndrome characterized by: (1) concern regarding a change in cognition reported by the individual, an informant, or a clinician; (2) objective evidence of impairment in one or more cognitive domains—such as memory, executive function, attention, language, or visuospatial skills—that exceeds age- and education-adjusted expectations; and (3) preservation of functional independence in daily activities, with only minimal inefficiencies. MCI was diagnosed when it was observed at two consecutive visits. Participants classified as having PD did not undergo further cognitive assessments. Participants without a dementia diagnosis continued to undergo routine cognitive assessments at scheduled intervals. Consequently, individuals who transitioned from MCI to PD during follow-up were not reassessed for MCI. This approach resulted in the number of participants classified under the composite outcome being lower than the sum of those with PD and MCI at baseline.

### 2.5. Statistical Analyses

Participants were categorized into two groups based on the presence of AF on their baseline or follow-up 12-lead ECG. Baseline characteristics were compared between the two groups using an independent sample *t*-test for continuous variables and the chi-square test for categorical variables.

Multivariable Cox proportional hazards models were used to examine the association between time-dependent AF status and the incidence of cognitive outcomes (PD, MCI, and their composite outcome (PD/MCI), separately). The first model was adjusted for age, sex, race, education level, and treatment assignment. The second model incorporated additional adjustments for baseline SBP, smoking status, alcohol consumption, history of cardiovascular disease (CVD), number of antihypertensive medications, serum creatinine levels, total cholesterol levels, and statin use. In an additional model, we also adjusted for incident stroke occurring during follow-up. Participants were censored either at the time of diagnosis of cognitive outcomes (PD, MCI, or the composite outcome) or at the conclusion of their last follow-up visit, whichever occurred first.

As a sensitivity analysis, we excluded participants diagnosed with MCI within the first two years of follow-up to mitigate potential bias from undetected prevalent MCI at baseline, given that the SPRINT MIND did not adjudicate baseline MCI status. We also conducted a competing risk analysis to account for the competing risk of death.

The consistency of the associations between time-dependent AF and cognitive outcomes among SPRINT pre-specified subgroups (age [<75 versus ≥75 years], sex, race [Black versus non-Black], SBP tertiles [≤132, >132 to <145, ≥145 mm Hg], prior CVD, and prior chronic kidney disease [defined as an estimated glomerular filtration rate of <60 mL/min per 1.73 m^2^ of body-surface area]) was assessed with a likelihood-ratio test for the interaction with the use of Hommel adjusted. The models were adjusted in a manner similar to Model 2 in the main analysis.

All analyses were performed using R software (version 4.1.1, PBC, Boston, MA, USA) and SPSS software (version 27, IBM Corp., Armonk, NY, USA). Statistical significance was set at a two-sided *p*-value threshold of < 0.05.

## 3. Results

A total of 8539 participants (mean age 67.9 years, 35.1% female) with available baseline and follow-up ECG and cognitive outcome data were included in our analysis. Of these, 264 participants had AF at baseline (*n* = 111; 47 in the standard arm and 64 in the intensive arm) or during the follow-up (*n* = 153; 84 in the standard arm and 69 in the intensive arm). Participants with AF were generally older, more likely to be male, of White ethnicity, and had a higher prevalence of CVD history. Additionally, they exhibited lower eGFR and lower total cholesterol levels (Table 1).

Over a median follow-up period of 5 years (interquartile range: 3.8–5.9 years), 318 PD, 625 MCI, and 849 composite endpoints of MCI/PDI occurred. In multivariable Cox regression models adjusted for sociodemographics and treatment assignment, AF as a time-dependent variable was significantly associated with an increased risk of PD, MCI, and a composite endpoint of MCI/PDI (Hazard Ratio (HR) (95% CI): 1.98 (1.19, 3.29), 1.64 (1.04, 2.57), and 1.74 (1.21, 2.50), respectively). These associations remained significant after further adjustment for CVD risk factors and potential confounders (HR (95% CI): 1.84 (1.09, 3.13), 1.59 (1.01, 2.53), and 1.63 (1.12, 2.38), respectively); Table 2.

Further adjustment for incident stroke did not materially impact the results (HR (95% CI): 1.71 (1.06, 2.91), 1.53 (0.96, 2.43), and 1.55 (1.06, 2.27)). In addition, excluding incident MCI occurring within the first two years of follow-up, AF as a time-dependent variable remained significantly associated with MCI (HR (95% CI): 1.69 (1.02, 2.82)) even after full adjustment for incident stroke (Supplemental Table S1). In additional analysis in which death was considered as a competing risk, AF as a time-dependent variable remained strongly associated with increased risk of PD, MCI and a composite end point of MCI/PDI (HR (95% CI): (1.95 (1.15, 3.32), 1.69 (1.06, 2.68), 1.73 (1.18, 2.52), respectively)) (Supplemental Table S2).

**Figure 1:**
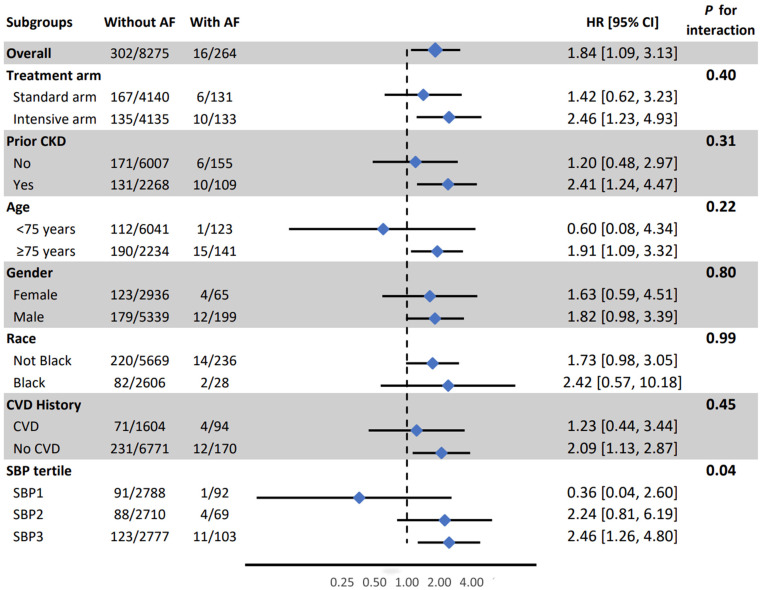
Association between time-dependent atrial fibrillation and probable dementia in pre-specified subgroups. AF: Atrial Fibrillation, CI: Confidence Interval, CKD: Chronic Kidney Disease, CVD: Cardiovascular Disease, HR: Hazard Ratio, SBP: Systolic Blood Pressure. The model was adjusted for age, sex, race, education, treatment assignment, systolic blood pressure, smoking, alcohol consumption, prior cardiovascular diseases, number of antihypertensive medications, serum creatinine, total cholesterol, and statin use.

**Figure 2:**
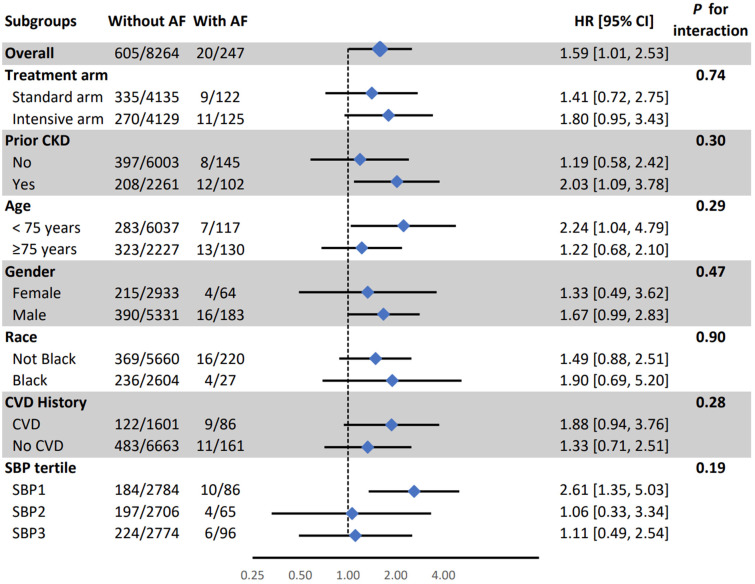
Association between time-dependent atrial fibrillation and mild cognitive impairment in pre-specified subgroups AF: Atrial Fibrillation, CI: Confidence Interval, CKD: Chronic Kidney Disease, CVD: Cardiovascular Disease, HR: Hazard Ratio, SBP: Systolic Blood Pressure. The model was adjusted for age, sex, race, education, treatment assignment, systolic blood pressure, smoking, alcohol consumption, prior cardiovascular diseases, number of antihypertensive medications, serum creatinine, total cholesterol, and statin use.

**Figure 3:**
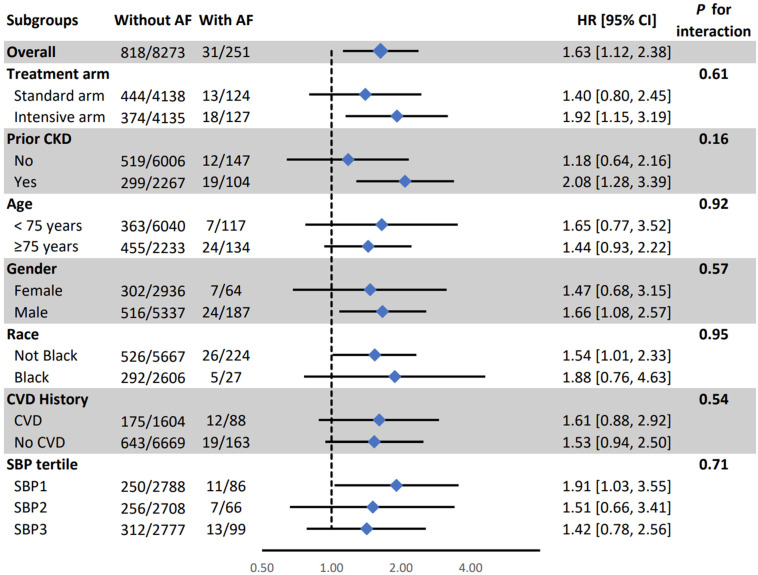
Association between time-dependent atrial fibrillation and a composite of probable dementia, mild cognitive impairment in subgroups.AF: Atrial Fibrillation, CI: Confidence Interval, CKD: Chronic Kidney Disease, CVD: Cardiovascular Disease, HR: Hazard Ratio, SBP: Systolic Blood Pressure. The model adjusted for age, sex, race, education, treatment assignment, systolic blood pressure, smoking, alcohol consumption, prior cardiovascular diseases, number of antihypertensive medications, serum creatinine, total cholesterol, and statin use.

In subgroup analysis, the association between AF and PD was weaker in the lowest SBP tertile than in other tertiles (HR (95% CI) for 1st, 2nd, and 3rd tertile: 036 (0.04, 2.60), 2.24 (0.81, 6.19), and 2.46 (1.26, 4.80), respectively; interaction *p*-value =0.04) (Figure 1). All other associations were consistent in the SPRINT pre-specified subgroups (Figure 1, Figure 2 and Figure 3).

## 4. Discussion

At present, there are no widely available effective treatments that favorably alter the natural history of cognitive decline and dementia, emphasizing the importance of identifying modifiable risk factors. In this analysis of the SPRINT trial, we found that AF was significantly associated with an increased risk of PD, MCI, and a composite endpoint of MCI/PDI outcomes. These associations were independent of baseline CVD risk factors or incident stroke during follow-up. In the subgroup analysis, the association between AF and PD was stronger among participants in the second and third SBP tertiles than among those in the first tertile. Collectively, our results emphasize the potential for early detection and management of AF in patients with hypertension to reduce the risk of cognitive impairment and underscore the importance of low SBP in reducing the impact of AF on the risk of cognitive decline. Further research is needed to investigate whether targeted interventions, such as rhythm control or anticoagulant therapy, can effectively mitigate the cognitive risks associated with AF.

Previous studies have reported inconsistent findings regarding the association between AF and cognitive impairment in the general population. For instance, an analysis from the Rotterdam Study, which examined the relationship between AF and incident dementia in white participants over a 20-year period, found no significant association between AF and dementia after adjusting for stroke [10]. Conversely, the Atherosclerosis Risk in Communities– Neurocognitive Study (ARIC-NCS), which evaluated a biracial population over a 20-year period, suggested that AF is significantly associated with both cognitive decline and incident dementia, even after adjusting for ischemic stroke [3]. Moreover, the Whitehall II study, which followed a cohort aged 45 to 85 years over 15 years, reported a significantly higher risk of incident dementia among AF participants, even after multivariable adjustment for potential confounders, including stroke [11]. The association between AF and dementia in the Whitehall II study was consistent in both older and younger age groups, and our findings are in agreement with the findings from the ARIC and Whitehall II studies. Racial diversity and age of the populations may explain the inconsistencies among different studies.

Although AF has been linked to an increased risk of cognitive impairment, decline, and dementia [12,13,14], its association with specific dementia subtypes remains unclear. While some studies have connected AF to various forms of dementia, including vascular, senile, mixed dementia, and Alzheimer’s disease [15,16], others have suggested a stronger correlation with Alzheimer’s disease, followed by vascular dementia [17]. However, the mechanisms underlying this association are not fully understood [18].

Some studies have attributed AF-related cognitive impairment to an elevated risk of stroke and TIA [19,20]. However, as our findings and the ARIC study show, this association persists even after excluding patients with stroke. A recent meta-analysis of over 2.8 million individuals further supports this, demonstrating AF’s consistent link between AF and cognitive impairment in the general population, stroke-free individuals, and post-stroke cohorts [21]. This implies that AF itself, beyond overt stroke, may drive cognitive decline through different mechanisms. One such mechanism could be a silent cerebral infarction [22,23]. The ARIC Study found that AF was associated with cognitive decline only in patients with MRI evidence of silent cerebral infarction [24]. Shared vascular risk factors may have contributed to this relationship. In our study, even after excluding diabetes and adjusting for other vascular risks (e.g., high SBP, dyslipidemia, and smoking), AF remained significantly associated with cognitive impairment. Additionally, beat-to-beat variability and reduced stroke volume in AF may cause cerebral hypoperfusion, leading to cerebral volume loss, white matter lesions, and small-vessel disease [25,26]. These pathological changes could further contribute to cognitive decline in patients with AF.

It is important to consider whether enhanced screening for AF and timely clinical interventions may reduce the risk of cognitive decline in hypertensive patients without a history of stroke or diabetes, who are at an elevated risk for both AF and cognitive impairment. One of the key modifiable risk factors in this population is blood pressure. In this context, a central finding of the SPRINT-MIND trial was that intensive blood pressure management significantly reduced the risk of MCI and the combined outcome of MCI and PD, but not PD alone. Moreover, intensive blood pressure treatment was associated with a 26% reduction in the incidence of AF. Although our interaction analysis did not reveal a statistically significant modifying effect of treatment assignment on the association between AF and cognitive outcomes, this finding should be interpreted with caution. The lack of significance may be due, at least in part, to the limited number of AF cases within certain treatment arms, which may have reduced the statistical power.

Recent evidence has also underscored the potential of sodium-glucose co-transporter 2 (SGLT2) inhibitors in reducing both AF incidence and cognitive decline. A meta-analysis of 52 randomized controlled trials demonstrated that SGLT2 inhibitors significantly lower the risk of new-onset AF, particularly in patients with heart failure with reduced ejection fraction (HFrEF) [27]. However, the observed benefits were less pronounced in individuals with hypertension and preserved ejection fraction, which are characteristics more representative of the SPRINT cohort.

Beyond cardiovascular effects, SGLT2 inhibitors have neuroprotective potential, with emerging evidence supporting their association with a reduced risk of cognitive impairment and dementia. Proposed mechanisms include improved glycemic control, reduced cerebrovascular risk, and attenuation of neuroinflammation. Notably, one large observational study demonstrated that even after adjusting for shared risk factors, such as stroke and other comorbidities, SGLT2 inhibitor use remained independently associated with a lower risk of both Alzheimer’s disease and vascular dementia [28].

Taken together, these findings suggest that SGLT2 inhibitors may offer dual benefits by lowering the incidence of both AF and cognitive decline. Future randomized clinical trials are warranted to evaluate the role of these agents in hypertensive patients without diabetes or a history of stroke. Such studies may help determine whether SGLT2 inhibitors can serve as a preventive strategy for cognitive impairment in individuals at risk of or already diagnosed with AF.

Beyond SGLT2 inhibitors, other cardiometabolic agents, such as statins, have also been hypothesized to play a role in preventing AF [29] and cognitive decline [30,31]. However, evidence regarding their effects on cognitive function remains inconclusive. While statins are well established for cardiovascular risk reduction, their impact on cognitive outcomes, particularly in elderly individuals without known atherosclerotic CVD, continues to be debated. Two systematic reviews found no consistent association between statin use and the risk of cognitive impairment or dementia. Furthermore, a large retrospective cohort analysis involving over 18,000 older adults without prior CVD or baseline cognitive impairment, followed for nearly five years, found no significant association between statin therapy and the incidence of dementia, MCI, or a decline in specific cognitive domains [30,31]. Ongoing randomized controlled trials, such as the PRagmatic EValuation of evENTs And Benefits of Lipid-lowering in oldEr adults (PREVENTABLE) (NCT04262206) and STAtins in Reducing Events in the Elderly (STAREE) (NCT02099123) studies, aim to definitively assess whether statins offer cognitive or functional benefits in primary prevention settings among older adults. The results of these trials will be critical in clarifying the role of statins in the preservation of cognitive function and guiding clinical decision-making in geriatric populations.

### Limitations and Strengths

Our study has some limitations that warrant consideration. The current analysis focuses exclusively on a population with hypertension, without a history of diabetes mellitus or prior stroke. Consequently, the generalizability of our findings to broader populations should be interpreted with caution. Second, our analysis was based on ECG-detected AF, which may underestimate the true burden of AF, particularly paroxysmal AF, which could be detected by long-term ECG recording. Nevertheless, undetected AF can only bias the results to null; hence, the reported associations in our analysis are conservative and could be stronger if undetected AF were included. In addition, we did not use self-reported history of prior AF diagnosis as a method of AF ascertainment. While this could affect external validity and complicate comparisons with studies that relied on self-report, self-reported AF is a subjective measure and may be prone to biases such as recall or information bias, increasing the risk of false positives. Although ECG may miss some cases of paroxysmal AF that could have been detected through patient history, any misclassification of true AF cases as non-AF would likely bias the results toward the null, making our findings conservative in that context, as previously mentioned. Third, dementia or MCI may develop several years after the onset of AF. A longer follow-up duration could potentially strengthen the observed association between AF and cognitive outcomes in this population. Fourth, although we adjusted for a comprehensive set of well-established covariates, including demographic factors, cardiovascular risk factors, and comorbidities, residual confounding by unmeasured risk factors remains a possibility. For example, we could examine the confounding impact of frailty, the mediating effect of anticoagulation therapy, or fluctuation in blood pressure over time. Finally, non-statistically significant findings in some subgroup or interaction analyses should be interpreted with caution, as these analyses may have been underpowered to detect modest effect modification.

The present study had several notable strengths. Cognitive outcomes were adjudicated by a committee of experts, and AF was assessed centrally by individuals blinded to the study outcomes. The study population comprised a distinct group of high-risk hypertensive patients without a history of stroke, addressing a critical gap in the literature. By excluding individuals with a history of stroke and diabetes and accounting for key cardiovascular risk factors, our study provides novel insights into the independent relationship between AF and cognitive impairment, minimizing confounding factors from cerebrovascular and CVD risk factors. Furthermore, we employed a time-dependent modeling approach for AF, allowing for a more accurate representation of its dynamic characteristics over time. The inclusion of subgroup analyses based on age, race, sex, treatment assignment, CVD history, and CKD further strengthens our study by offering a nuanced understanding of potential effect modifications across diverse populations.

## 5. Conclusions

AF is associated with an increased risk of cognitive impairment and dementia, independent of vascular risk factors and stroke in patients with hypertension but without diabetes. Further research is needed to explore the underlying mechanisms and to investigate whether targeted interventions, such as rhythm control or anticoagulation therapy, can effectively mitigate the cognitive risks associated with AF.

## Figures and Tables

**Table 1:** Baseline characteristics of the participants stratified by atrial fibrillation.

Variables *n* (%) or Mean ± SD	Total Participants (*n* = 8539)	With AF (*n* = 264)	Without AF (*n* = 8275)
Age (year)	67.9 ± 9.3	74.0 ± 8.6	67.7 ± 9.2
Female	3001 (35.1%)	65 (24.6%)	2936 (35.5%)
Race/Ethnicity			
Black	2504 (29.3%)	27 (10.2%)	2477 (29.9%)
Hispanic	883 (10.3%)	15 (5.7%)	868 (10.5%)
White	4997 (58.5%)	219 (83%)	4778 (57.7%)
Others	155 (1.8%)	3 (1.1%)	152 (1.8%)
Non-College graduate	5075 (59.4%)	143 (54.2%)	4932 (59.6%)
Intensive treatment arm	4268 (50.0%)	133 (50.4%)	4135 (50.0%)
Prior CVD	1698 (19.8%)	94 (35.6%)	1604 (19.4%)
Smoking history, *n* (%)			
Former	3662/8530 (42.9%)	163/264 (61.7%)	3499/8266 (42.3%)
Current	1095/8530 (12.8%)	16/264 (6.1%)	1079/8266 (13%)
Alcohol use	331/8534 (3.8%)	5 (1.9%)	326 (3.9%)
SBP	139.5 ± 15.5	141.5 ± 17.5	139.4 ± 15.4
SBP tertiles			
≤132 mm Hg	2880 (33.7%)	92 (34.8%)	2788 (33.7%)
>132 to <145 mm Hg	2779 (32.5%)	69 (26.1%)	2710 (32.7%)
≥145 mm Hg	2880 (33.7%)	103 (39.0%)	2777 (33.6%)
No. of BP lowering drugs	1.83 ± 1.03	2.30 ± 0.99	1.82 ± 1.03
eGFR	71.8 ± 20.4	65.7 ± 18.1	72 ± 20.4
Serum creatinine	1.07 ± 0.33	1.11 ± 0.30	1.07 ± 0.33
Total cholesterol	190.0 ± 41.1	176.9 ± 38.5	190.5 ± 41.1
Statin use, *n* (%)	3736/8486 (44%)	141/262 (53.8%)	3595/8224 (43.7%)
HDL-Cholesterol	52.7 ± 14.3	52.1 ± 13.9	52.8 ± 14.3
BMI	29.8 ± 5.7	30.2 ± 5.6	29.8 ± 5.7

AF, atrial fibrillation; BMI: Body Mass Index, DBP: Diastolic Blood Pressure, SBP: Systolic Blood Pressure, CVD = Cardiovascular Disease; eGFR, Estimated Glomerular Filtration Rate, HDL: High-Density Lipoprotein.

**Table 2:** Association of time-dependent atrial fibrillation with cognitive outcomes in the SPRINT trial.

Outcome	Events/Participants *n* (%)		Model 1		Model 2	
	With AF (*n* = 264)	Without AF (*n* = 8275)	Hazard Ratio (95% CI)	*p*-Value	Hazard Ratio (95% CI)	*p*-Value
Probable Dementia (PD)	16/264 (6.1%)	302/8275 (3.6%)	1.98 (1.19, 3.29)	0.007	1.84 (1.09, 3.13)	0.02
Mild Cognitive Impairment (MCI)	20/247 (8.1%)	605/8264 (7.3%)	1.64 (1.04, 2.57)	0.03	1.59 (1.01, 2.53)	0.04
Composite MCI/PD	31/251 (12.3%)	818/8273 (9.8%)	1.74 (1.21, 2.50)	0.002	1.63 (1.12, 2.38)	0.009

CI: Confidence Interval, *n*: Number, SPRINT: Systolic Blood Pressure Intervention Trial. Model 1 was adjusted for age, sex, race, education, and treatment assignment. Model 2 was adjusted for Model 1 plus systolic blood pressure, smoking, alcohol consumption, prior cardiovascular diseases, number of antihypertensive medications, serum creatinine, total cholesterol, and statin use.

## Data Availability

All data in this manuscript were sourced from the National Institutes of Health Biologic Specimen and Data Repository Information Coordinating Center. Access to these resources is available at https://biolincc.nhlbi.nih.gov/studies/sprint/ (accessed on 20 December 2024).

## Supplementary Materials

The following supporting information can be downloaded at: https://www.mdpi.com/article/10.3390/jcm14134791/s1, Table S1. Association of time-dependent AF with MCI excluding the first two years of incident mild cognitive impairment (MCI); Table S2. Association of time-dependent atrial fibrillation with cognitive outcomes in the SPRINT trial in participants accounting for competing risk of death.
