# Supplementary material for: Association of Atrial Fibrillation with Incident Probable Dementia and Cognitive Impairment in the Systolic Blood Pressure Intervention Trial (SPRINT)
Source: J Clin Med. 2025 Jul 7;14(13):4791. doi: 10.3390/jcm14134791 (PMC12250728; doi:10.3390/jcm14134791)
Supplement: Supplementary file 1 [file jcm-14-04791-s001.zip › jcm-3700614-supplementary.pdf]

Supplementary Table S1. Association of time-dependent AF with MCI excluding the first two years of incident mild cognitive impairment (MCI)

**Outcome**	**Events/participants n (%)**		**Model 1**		**Model 2**		**Model 3**	
	**With AF**	**Without AF**	**Hazard ratio (95% CI)**	*p-value*	**Hazard ratio (95% CI)**	*p-value*	**Hazard ratio (95% CI)**	*p-value*
**MCI**	16/243	416/8075	1.73 (1.04, 2.86)	0.03	1.79 (1.07, 2.97)	0.02	1.69 (1.02, 2.82)	0.04

CI: Confidence Interval, n: Number, SPRINT: Systolic Blood Pressure Intervention Trial
Model 1 adjusted for age, sex, race, education, and treatment assignment.
Model 2 adjusted for model 1 plus systolic blood pressure, smoking, alcohol consumption, prior cardiovascular diseases, number of antihypertensive medications, serum creatinine, total cholesterol, and statin use.
Model 3 adjusted for model 2 plus the incident stroke.

Supplementary Table S2. Association of time-dependent atrial fibrillation with cognitive outcomes in the SPRINT trial in participants accounting for competing risk of death.

Outcome	Events/participants n (%)		Model 1		Model 2	
	**With AF (n=264)**	**Without AF (n=8275)**	**Hazard ratio (95% CI)**	*p-value*	**Hazard ratio (95% CI)**	*p-value*
**Probable Dementia (PD)**	16/256 (6.2%)	293/8147 (3.6%)	2.09 (1.26, 3.48)	0.004	1.95 (1.15, 3.32)	0.012
**Mild Cognitive Impairment (MCI)**	20/239 (8.3%)	592/8136 (7.3%)	1.72 (1.10, 2.70)	0.017	1.69 (1.06, 2.68)	0.026
**Composite MCI/PD**	31/243 (12.7%)	799/8145 (9.8%)	1.83 (1.27, 2.63)	0.001	1.73 (1.18, 2.52)	0.004

CI: Confidence Interval, n: Number, SPRINT: Systolic Blood Pressure Intervention Trial
Model 1 adjusted for age, sex, race, education, and treatment assignment.
Model 2 adjusted for model 1 plus systolic blood pressure, smoking, alcohol consumption, prior cardiovascular diseases, number of antihypertensive medications, serum creatinine, total cholesterol, and statin use.
